# Kinetic modeling of countercurrent saccharification

**DOI:** 10.1186/s13068-019-1517-5

**Published:** 2019-07-11

**Authors:** Chao Liang, Chao Gu, M. Nazmul Karim, Mark Holtzapple

**Affiliations:** 1 Department of Chemical Engineering, Texas A&M University, College Station, TX 77843-3122 USA; 2 Texas A&M Institute for Neuroscience, Texas A&M University, College Station, TX 77843-3122 USA

**Keywords:** Countercurrent, Saccharification, Simulation, CPDM, Sugar

## Abstract

**Background:**

Countercurrent saccharification is a promising way to minimize enzyme loading while obtaining high conversions and product concentrations. However, in countercurrent saccharification experiments, 3–4 months are usually required to acquire a single steady-state data point. To save labor and time, simulation of this process is necessary to test various reaction conditions and determine the optimal operating point. Previously, a suitable kinetic model for countercurrent saccharification has never been reported. The Continuum Particle Distribution Modeling (CPDM) satisfactorily predicts countercurrent fermentation using mixed microbial cultures that digest various feedstocks. Here, CPDM is applied to countercurrent enzymatic saccharification of lignocellulose.

**Results:**

CPDM was used to simulate multi-stage countercurrent saccharifications of a lignocellulose model compound (α-cellulose). The modified HCH-1 model, which accurately predicts long-term batch saccharification, was used as the governing equation in the CPDM model. When validated against experimental countercurrent saccharification data, it predicts experimental glucose concentrations and conversions with the average errors of 3.5% and 4.7%, respectively. CPDM predicts conversion and product concentration with varying enzyme-addition location, total stage number, enzyme loading, liquid residence time (LRT), and solids loading rate (SLR). In addition, countercurrent saccharification was compared to batch saccharification at the same conversion, product concentration, and reactor volume. Results show that countercurrent saccharification is particularly beneficial when the product concentration is low.

**Conclusions:**

The CPDM model was used to simulate multi-stage countercurrent saccharification of α-cellulose. The model predictions agreed well with the experimental glucose concentrations and conversions. CPDM prediction results showed that the enzyme-addition location, enzyme loading, LRT, and SLR significantly affected the glucose concentration and conversion. Compared to batch saccharification at the same conversion, product concentration, and reactor volume, countercurrent saccharification is particularly beneficial when the product concentration is low.

## Background

Enzymatic saccharification of non-food biomass, such as lignocellulose, can produce sugars. Sugars are a common feedstock for bioethanol, which can be substituted for transportation fuels [1–3]. As substrate is hydrolyzed, traditional batch enzymatic saccharification cannot fully use substrate because biomass becomes less reactive [4, 5], while the enzymes become increasingly inhibited by accumulated product; therefore, high enzyme loadings are usually required to reach high conversions [6, 7]. To overcome these obstacles, countercurrent enzymatic saccharification was developed, where the least reactive biomass contacts the lowest glucose concentration and the product is removed continuously from the system, thus reducing product inhibition [8–10]. This approach more fully utilizes enzymes and therefore reduces the enzyme loadings and lowers the cost of sugar and biofuel production.

**Fig. 1:**
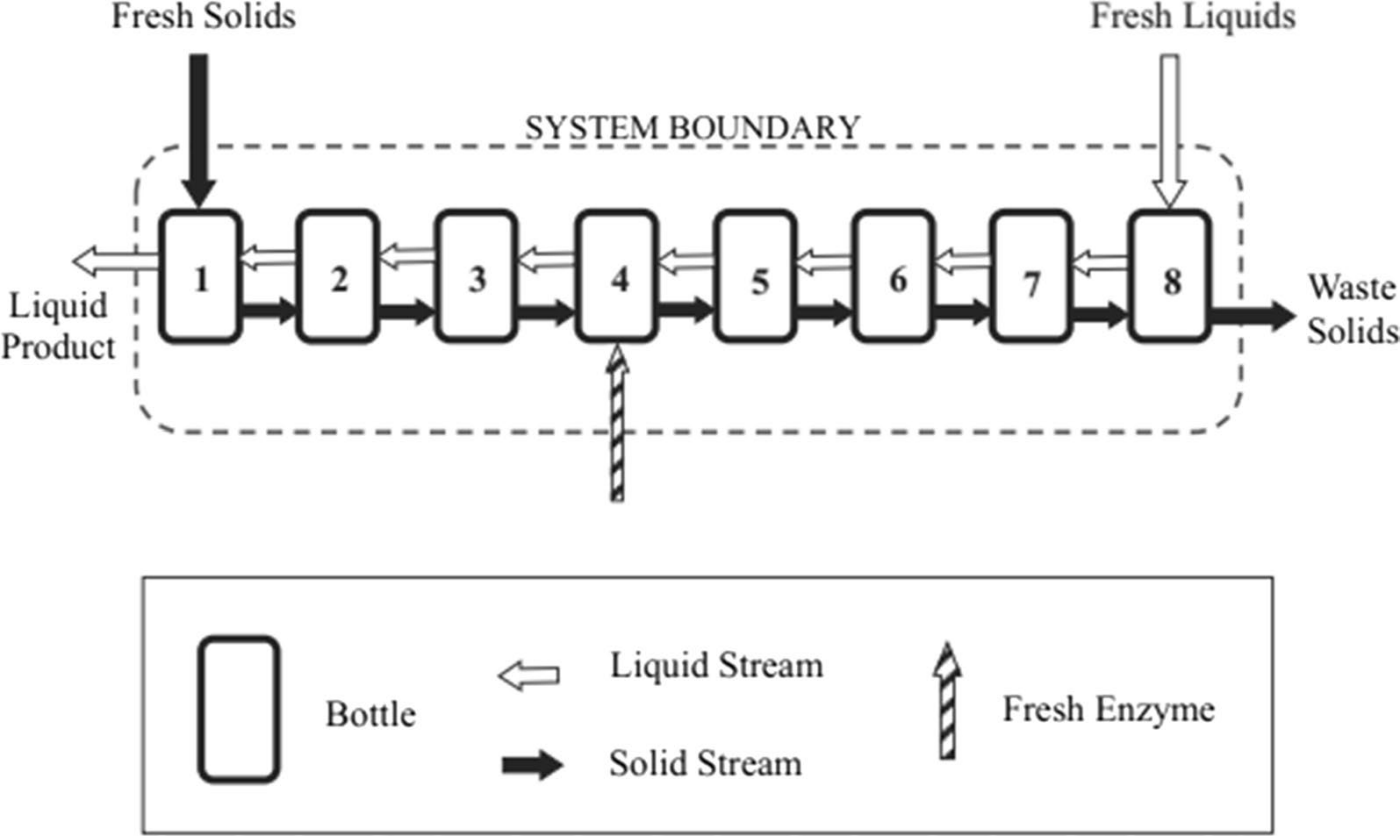
System diagram of countercurrent saccharification (Zentay et al. [8])

Zentay et al. [8] performed multi-stage semi-continuous countercurrent saccharifications using lignocellulose model compound (α-cellulose) and commercial cellulase cocktail Novozymes CTec2 (Fig. 1). Compared to standard (5-day) batch saccharification, to reach a given glucose conversion, countercurrent saccharification reduced enzyme loadings by 8–20.5 times. The great reduction resulted from the inherent benefits of countercurrent saccharification as well as a longer residence time. Lonkar et al. [9] and Liang et al. [10] continued this study with pretreated biomass. To achieve the same glucan conversion, as compared to batch, countercurrent saccharification reduced enzyme loadings up to 1.6 and 1.9 times with lime-pretreated and lime + shock-treated corn stover, respectively.

Liang et al. [10] showed that adding a volatile antimicrobial prevented contamination even when purposely assaulted with an active culture of soil-derived microorganisms; thus, extremely long residence times can be employed. The volatile antimicrobial can be removed from the product sugars and hence is recycled. According to Liang et al. [10], this technology can produce sugars for about $0.26 to $0.35/kg including the cost of feedstock, pretreatment, and enzyme. As a reference point, the average price of sugar has been about $0.40/kg during the past 10 years [11].

In countercurrent saccharification experiments, 3–4 months are usually required to acquire a single steady-state data point. To save labor and time, simulation of this process is necessary to test various reaction conditions and determine the optimal operating point. Previously, a suitable kinetic model for countercurrent saccharification has never been reported.

Loescher [12] developed Continuum Particle Distribution Modeling (CPDM) theory and derived it for various reactor configurations [batch, fed batch, continuous stirred-tank reactor (CSTR), plug-flow reactor (PFR), countercurrent and cocurrent CSTR cascades, PFR-CSTR cascades, and CSTR-PFR cascades]. Previous studies [12–16] showed that the CPDM model satisfactorily predicts countercurrent fermentation using mixed microbial cultures that digest various feedstocks. Here, CPDM is applied to countercurrent enzymatic saccharification of lignocellulose.

In this study, the CPDM model was used to simulate multi-stage countercurrent saccharification. α-Cellulose was used for modeling because of its simpler composition compared to real-world lignocellulose, which contains lignin. Lignin is known to bind cellulase enzymes non-productively [17], which complicates the kinetic modeling. The predicted glucose concentrations and conversions were compared with experimental data and the average errors were calculated. In addition, the model estimated the effects of enzyme-addition location, total stage number, enzyme loading, liquid residence time (LRT), and solids loading rate (SLR) on conversion and product concentration, thus allowing the benefits of countercurrent saccharification over batch to be quantified.

## Materials and methods

### Materials

The substrate used for all experiments was α-cellulose (Sigma Aldrich, C8002-5KG). Compositional analysis showed that the substrate contained glucan 78.5% and xylan 14.4% [8]. The enzyme used for all experiments was Novozymes Ctec2 (lot # VCPI 0007), a blend of aggressive cellulases with high levels of β-glucosidases and hemicellulases [8, 9]. The protein concentration of the enzyme solution was determined to be 294 mg protein/mL with Pierce BCA assay [8]. To maintain relatively high enzyme activity, citrate buffer (0.1 M, pH 4.8) was used in all experiments. To prevent microbial growth, an antibiotic cocktail [tetracycline 10 g/L 70% ethanol and cycloheximide 10 g/L deionized (DI) water] was used in all experiments.

### Countercurrent saccharification

Experimental data of countercurrent saccharification were obtained from Zentay et al. [8]. In the experiments, two trains were performed with enzyme loadings of 2 and 5 mg protein added/g dry biomass added (mg/g), respectively.

To begin the countercurrent experiment, all the stages (Nalgene centrifuge bottles, 1 L, Fisher catalog # 05-562-26) were loaded with 250 g materials, including 25 g dry α-cellulose, 125 mL citrate buffer, 2 mL tetracycline solutions, 1.5 mL cycloheximide solutions, and given amounts of enzyme solution and DI water (Note: the density of all liquid materials was assumed to be 1 g/cm^3^). The bottles were placed in 4-in-ID PVC pipes and axially rotated at 2 rpm by a Wheaton Roller Apparatus located in the custom-made incubator at 50 °C. The transfers were performed every 48 h. In every transfer, each bottle was centrifuged to achieve phase separation of liquid and solid wet cake (70–80% moisture content). The mass of each phase and the pH of the liquid phase were measured. A liquid sample (1 mL) was taken from every bottle and analyzed by HPLC (Agilent 1260 Infinity) to determine when the system reached steady state. All the liquid was moved from “back” to “front” and the solid phases were moved in the opposite direction (Fig. 1). The amount of solid phase transferred was calculated to ensure each stage had the same amount of wet cake (Train 1: 85 g; Train 2: 80 g) after each transfer. Then, 10 g dry biomass was loaded in Stage 1 and 90 mL liquid consisting of 50 mL citrate buffer and 40 mL DI water was added to Stage 8. Antibiotic solutions (0.4 mL tetracycline solution and 0.3 mL cycloheximide solution) were introduced to every stage and the desired amount of enzymes was added to a specific location (Train 1: 2 mg/g to Stage 4; Train 2: 5 mg/g to Stage 5). When the sugar concentrations from each stage did not show significant change over a relatively long time (e.g., 15 days), the system was determined to reach steady state [8–10, 18]. Table 1 summarizes the operating parameters of the two trains. More details of the experiments and mass balance calculations are shown in Zentay et al. [8].

**Table 1:** Operating parameters of eight-stage countercurrent saccharification experiments

Train	1	2
Substrate	α-Cellulose	α-Cellulose
Added solids in every transfer (g)	10	10
Added liquid in every transfer (mL)	90	90
Enzyme loading (mg/g)	2	5
Total reaction time (days)	24	42
Transfer frequency	Every 48 h	Every 48 h
Incubation temperature (°C)	50	50
Enzyme-addition location (stage number)	4	5

The conversion was determined as the ratio of glucose exiting the countercurrent system to the equivalent glucose entering the system in each transfer (Note: Cellobiose was not considered in this study because the enzyme CTec2 contains a high level of β-glucosidase that could rapidly convert the produced cellobiose into glucose). The glucose exiting the system was the summation of glucose exiting from Stages 1 and 8, and glucose in liquid samples from all eight stages. The equivalent glucose entering the system was from the substrate added to the first stage (Eq. 1).

1 $${\text{Conversion }}\left( \% \right) = \frac{{{\text{glucose}}\;{\text{exiting}}\;{\text{from}}\;{\text{first}}\;{\text{and}}\;{\text{last}}\;{\text{stages}} \;\left( {\text{g}} \right) + {\text{glucose}}\;{\text{in}}\;{\text{liquid}}\;{\text{samples}}\;{\text{from}}\;{\text{all}}\;{\text{stages}} \;\left( {\text{g}} \right)}}{{{\text{equivalent}}\;{\text{glucose}}\;{\text{in}}\;{\text{cellulose}}\;{\text{feed}}\; \left( {\text{g}} \right) }} \times 100\% .$$

### Continuum Particle Distribution Model

In CPDM, a “continuum particle” is defined as 1 g of solids in the initial unreacted state and is representative of the substrate [12, 13, 16]. This model tracks the reaction progress of the continuum particle as it transfers through the stage, digests, and releases products [19]. Conversion of the particle from 0 to 1 is divided into a given number of intervals. A conversion distribution function (implicitly defined by Eq. 2) is used to express the number of continuum particles in each specific conversion interval at a particular reaction time.

2 $$n_{0} = \mathop \int \limits_{0}^{1} \hat{n}\left( x \right){\text{d}}x$$

where $\hat{n}\left( x \right)$ is the particle conversion distribution function and $n_{0}$ is the initial particle concentration (particle/L).

Equation 3 relates the total reaction rate (*r*_t_) with the reaction rate at a given conversion (*r*) as a function of particle conversion (*x*), product concentration (*G*_1_), enzyme concentration (*E*), and particle conversion distribution $\hat{n}\left( x \right),$ which contains information about substrate concentrations (*G*_*x*_) and conversions (*x*). (Note: The CPDM model was previously used to simulate countercurrent fermentation only. The governing rate equation used in the CPDM model was for batch fermentation. To simulate countercurrent saccharification, in this paper, the governing rate equation of the CPDM model was changed).

3 $$r_{\text{t}} = \int\limits_{0}^{1} {r\left( {\hat{n}\left( x \right),\;x,\;\left[ {G_{1} } \right],\left[ E \right]} \right)} \,{\text{d}}x$$

where *r*_t_ is the total reaction rate (g/(L d)), *r* is the reaction rate at a given conversion (g/(L d)), *G*_1_ is the glucose concentration (g/L), *E* is the (native) enzyme concentration (g/L), and *x* is the substrate conversion (0–1).

To get a satisfactory prediction, the governing rate equation $\left( {r\left( {\hat{n}\left( x \right), x,\left[ {G_{1} } \right],\left[ E \right]} \right)} \right)$ employed in the CPDM model should accurately describe batch enzymatic hydrolysis under various reaction conditions. The HCH-1 model, proposed by Holtzapple et al. [20], is a generalized mechanistic model for cellulose hydrolysis. Compared to the classic Michaelis–Menten model, the HCH-1 model includes non-competitive inhibition and an added parameter $\varepsilon$ that describes the number of reactive sites covered by the enzymes [20, 21]. Previous studies [21] showed that the HCH-1 model could predict short-term (initial-rate) enzymatic hydrolysis with high accuracy and better than other models that appeared in the literature. Liang et al. [22] modified the HCH-1 model to extend its application to long-term (> 48 h) batch enzymatic hydrolysis. Comparison of mechanistic models under various reaction conditions showed that the modified HCH-1 model provided the best fit to experimental data of enzymatic hydrolysis of α-cellulose [21, 22]. Therefore, the modified HCH-1 model (Eq. 4, parameter values from [22]) was used as the governing rate equation in the CPDM model.

4 $$\begin{aligned} \frac{{\left. {{\text{d}}[G_{1} } \right]}}{{{\text{d}}t}} & = \frac{{\kappa \left[ {G_{x} } \right]\left[ E \right]i}}{{\alpha + \varphi \left[ {G_{x} } \right] + \varepsilon \left[ E \right]}} \\ i & = \frac{1}{{\left. {1 + \beta_{1} [G_{1} } \right]}} \\ \varphi & = \frac{{\left[ {G_{x} } \right] - \alpha - \varepsilon \left[ E \right] + \sqrt {\left( {\left[ {G_{x} } \right] - \alpha - \varepsilon \left[ E \right]} \right)^{2} + 4\alpha \left[ {G_{x} } \right]} }}{{2\left[ {G_{x} } \right]}} \\ - \frac{{\left. {{\text{d}}[E} \right]}}{{{\text{d}}t}} & = 0.023\left[ E \right] - 0.174\left( {\left[ {E_{0} } \right] - \left[ E \right]} \right)\left[ {E_{0} } \right] \\ \kappa & = \frac{{k_{3} }}{{\left( {1 + x^{{k_{4} }} } \right)^{{k_{5} }} }} + k_{6} \\ \alpha & = \frac{{a_{1} \left[ {G_{1} } \right]}}{{\left[ E \right]\left( {1 + \exp \left( { - a_{2} x + a_{3} } \right)} \right)}} \\ \end{aligned}$$

where *G*_*x*_ is the substrate concentration (unreacted, equivalent to glucose, g/L); *E*_0_ is the initial enzyme protein concentration (g/L); *α* is the lumped adsorption constant (g/L); *a*_1_, *a*_2_, and *a*_3_ are the parameters related to adsorption constant (*a*_1_ = 1.68 g/L, *a*_2_ = 31.15, and *a*_3_ = 2.85); *κ* is the lumped kinetic constant (h^−1^); *k*_3_, *k*_4_, *k*_5_, and *k*_6_ are the parameters related to kinetic constant (*k*_3_ = 84.75 h^−1^, *k*_4_ = 2.58, *k*_5_ = 26.36, and *k*_6_ = 38.50 h^−1^); *β*_1_ is the glucose binding constant (L/g, *β*_1_ = 0.043 L/g); $\varepsilon$ is the number of cellulose sites covered by adsorbed or complexed enzyme (dimensionless, $\varepsilon$ = 5.52 × 10^−5^); *i* is the fraction of total enzyme that is active (dimensionless); and $\varphi$ is the fraction of total cellulose sites that are free (dimensionless).

In the simulation of countercurrent saccharification, all stages were set to have the same initial conditions identical to the experiments (Table 1). Combining Eqs. 2 and 4, the reaction rate *r* of each conversion interval was calculated. Using Eq. 3, the total reaction rate $r_{t}$ of each stage was calculated and the glucose concentration of each stage after 48-h reaction was obtained. Accordingly, the conversions of the continuum particles were changed from the previous conversion intervals to the higher intervals. Then, just like the experiments, a 1-mL liquid sample was taken from each stage; specific amounts of solids and liquid phases were transferred between stages; solids and liquids were removed from the last and first stages, respectively; and fresh solids, liquid, and enzymes were added to the system. The conversion distribution of continuum particles and glucose concentration of each stage were further changed based on the transfer. Afterward, the next 48-h reaction started. The previous steps were repeated until the total reaction time was reached. Using Eq. 1, the conversion of the countercurrent system was calculated.

### Enzyme stability

The operating time of the countercurrent experiments was usually longer than 1 month. To improve the countercurrent saccharification model, it is necessary to determine enzyme stability over a relatively long time. Rosales-Calderon et al. [23] showed that the soluble protein concentration of a mixture of glucanase and β-glucosidase dropped significantly after incubating at 50 °C for 4 days and hypothesized that the enzyme proteins suffered a structural change, which led to protein aggregation and precipitation. Based on this hypothesis, Liang et al. [22] measured the stability of CTec2 by quantifying soluble protein concentration over the course of 20 days. Results showed that soluble CTec2 protein concentration dropped up to 26% after 20-day incubation at 50 °C. Equation 5 (proposed by Rosales-Calderon et al. [23], parameter values from Liang et al. [22]) was used to model the stability of CTec2 successfully. To predict active enzyme concentration in the countercurrent process accurately, Eq. 5 was incorporated into the simulation of countercurrent saccharification. It should be noted that in batch simulation, Eq. 5 was part of the modified HCH-1 model. Here, it is incorporated into the CPDM model independently instead of being included in the governing equation because the addition locations of substrate and enzymes were different in the countercurrent system.

5 $$- \frac{{\left. {{\text{d}}[E} \right]}}{{{\text{d}}t}} = 0.023\left[ E \right] - 0.174\left( {\left[ {E_{0} } \right] - \left[ E \right]} \right)\left[ {E_{0} } \right].$$

### Enzyme distribution

In every transfer of the countercurrent experiment, solid and liquid phases were separated by a centrifuge and transferred in the opposite direction. As the liquid/solid phases moved, the enzymes suspended in the liquid phase or absorbed on the solid phase would move with them. To predict accurately the enzyme concentration of each stage after every transfer, it is important to determine the distribution of enzymes between the two phases. Kumar and Wyman [24] showed that glucose addition and enzyme dosage can affect the fraction of cellulase adsorption. In this study, glucose concentrations ranging from 0 to 93.35 g/L and enzyme loadings ranging from 1 to 5 mg/g were tested.

In the enzyme distribution experiments, the desired amounts of glucose, enzyme solution, and DI water together with 25 g α-cellulose, 125 mL citrate buffer, and 3.5 mL antibiotic solutions were added to a 1-L centrifuge bottle (total 250 g). Control experiments were also performed, which had the same loadings, but without substrate. To avoid hydrolysis, the loaded bottles were placed in the refrigerator (4 °C). After equilibration overnight, the bottles were centrifuged to separate solid and liquid phases. The protein concentrations of supernatants were measured by the Bradford protein assay and the glucose concentrations of supernatants were analyzed by an HPLC that was equipped with a refractive index detector, autosampler, a pair of de-ashing guard columns (Bio-Rad Micro-Guard de-ashing cartridges, 30 mm × 4.6 mm), and an HPLC carbohydrate analysis column (BioRad Aminex HPX-87P, 300 mm × 7.8 mm). The fraction of enzyme absorbed on the solid phase was expressed by Eq. 6.

6 $${\text{Enzyme}}\;{\text{absorbed}}\; \left( {\text{fraction}} \right) = 1 - \frac{{{\text{protein}}\;{\text{concentration}}\;{\text{of}}\;{\text{supernatant}}\;{\text{in}}\;{\text{test}}\;{\text{experiment}}}}{{{\text{protein}}\;{\text{concentration}}\;{\text{of}}\;{\text{supernatant}}\;{\text{in}}\;{\text{corresponding}}\;{\text{control}}\;{\text{experiment}}}}.$$

### Reactor volume calculation

The volume of the batch reactor was determined as follows:

7 $${\text{Batch}} \;{\text{reactor}}\;{\text{volume}}\; \left( {\text{L}} \right) = \frac{{{\text{Glucose}}\;{\text{production}}\;{\text{rate}}\, \left( {\frac{{{\text{g}}\;{\text{glucose}}}}{\text{day}}} \right) \times {\text{Batch}} \;{\text{residence}}\;{\text{time }} \left( {\text{day}} \right)}}{{{\text{Product}}\;{\text{concentration}} \,\left( {\frac{{{\text{g}}\;{\text{glucose}}}}{{{\text{L}}\;{\text{solution}}}}} \right) \times {\text{Ratio}}\;A\, \left( {\frac{{{\text{L}}\; {\text{solution}}}}{{{\text{L}}\;{\text{slurry}}}}} \right)}}$$

where Ratio *A* is the volume of sugar solution per the measured volume of the slurry before reaction. For example, if the glucose concentration of the product is 100 g/L, the density of the solution is approximately 1040 g/L solution [25]. In 1 L of sugar solution, there are 100 g of glucose and 940 g water. To make that amount of glucose requires 90 g cellulose and 10 g of water; therefore, the slurry would be composed of 90 g cellulose and 950 g water. Assuming the density of solid is 1 g/mL, then Ratio *A* is 0.962.

The volume of the countercurrent reactor was determined as follows:

8 $$\begin{aligned} & {\text{Countercurrent}}\;{\text{reactor}}\;{\text{volume}} \;\left( {\text{L}} \right) \\ & \quad = {\text{Glucose}}\;{\text{production}}\;{\text{rate}}\; \left( {\frac{{{\text{g}}\; {\text{glucose}}}}{\text{day}}} \right) \times {\text{Ratio}} \; B\, \left( {\frac{{{\text{g}}\; {\text{water}}}}{{{\text{g}}\;{\text{glucose}}}}} \right) \\ & \quad \quad \times {\text{Water}}\;{\text{density}} \left( {\frac{{{\text{L}}\;{\text{water}}}}{{1000 \; {\text{g}}\;{\text{water}}}}} \right) \times {\text{Ratio}} \;C \left( {\frac{{{\text{L}}\;{\text{slurry}}}}{{{\text{L}}\;{\text{water}}}}} \right) \\ & \quad \quad \times \;{\text{Liquid}}\;{\text{residence}}\;{\text{time }}\left( {\text{day}} \right) \\ \end{aligned}$$

where Ratio *B* is the ratio of the water mass to glucose mass at a specific glucose concentration, and Ratio *C* is the ratio of the initial total working volume to the loaded liquid volume. For example, if the solid concentration in the reactor is 250 g solids/L liquid, then Ratio *C* is the volume of 250 g solids + 1000 g water divided by the volume of 1000 g water. Assuming the density of solid is 1 g/mL, then Ratio *C* is 1.25.

In this study, to achieve a fair comparison, the batch reactor volume was considered identical to the continuous plug-flow reactor volume.

9 $$\begin{aligned} & \frac{{{\text{Glucose}}\;{\text{production}}\;{\text{rate}} \;\left( {\frac{{{\text{g}}\;{\text{glucose}}}}{\text{day}}} \right) \times {\text{Batch}}\;{\text{residence}}\;{\text{time }}\left( {\text{day}} \right)}}{{{\text{Product}}\;{\text{concentration}}\; \left( {\frac{{{\text{g}}\;{\text{glucose}}}}{{{\text{L}}\;{\text{solution}}}}} \right) \times {\text{Ratio }} A \;\left( {\frac{{{\text{L}}\;{\text{solution}}}}{{{\text{L}}\;{\text{slurry}}}}} \right)}} \\ & \qquad = {\text{Glucose}}\;{\text{production}}\;{\text{rate}}\; \left( {\frac{{{\text{g}}\;{\text{glucose}}}}{\text{day}}} \right) \times {\text{Ratio }} B \,\left( {\frac{{{\text{g}}\;{\text{water}}}}{{{\text{g}}\;{\text{glucose}}}}} \right) \\ & \qquad \quad \times {\text{Water}}\;{\text{density }} \left( {\frac{{{\text{L}}\;{\text{water}}}}{{1000 \; {\text{g}}\;{\text{water}}}}} \right) \times {\text{Ratio}}\, C\, \left( {\frac{{{\text{L}}\;{\text{slurry}}}}{{{\text{L}}\;{\text{water}}}}} \right) \\ & \qquad \quad \times \,{\text{Liquid}}\;{\text{residence}}\;{\text{time}} \,\left( {\text{day}} \right). \\ \end{aligned}$$

The glucose production rates of batch and countercurrent saccharifications were set equal, then,

10 $${\text{Liquid}}\;{\text{residence}}\;{\text{time }}\left( {\text{day}} \right) = \frac{{{\text{Batch}}\;{\text{residence}}\;{\text{time }}\left( {\text{day}} \right)}}{{{\text{Product}}\;{\text{concentration }}\;\left( {\frac{{{\text{g}}\;{\text{glucose}}}}{{{\text{L}}\;{\text{solution}}}}} \right) \times {\text{Ratio}} \;A\, \left( {\frac{{{\text{L}}\;{\text{solution}}}}{{{\text{L}}\;{\text{slurry}}}}} \right) \times {\text{Ratio}} \;B \left( {\frac{{{\text{g}}\;{\text{water}}}}{{{\text{g}}\;{\text{glucose}}}}} \right) \times {\text{Water}}\;{\text{density }} \left( {\frac{{{\text{L}}\;{\text{water}}}}{{1000 \; {\text{g}}\,{\text{water}}}}} \right) \times {\text{Ratio}}\, C\, \left( {\frac{{{\text{L}}\;{\text{slurry}}}}{{{\text{L}}\;{\text{water}}}}} \right)}}.$$

The denominator has typical values of 1 to 1.25.

Equation 10 specifies the required LRT for the countercurrent system that has the same volumetric productivity (g glucose/(L reactor day)) as the batch reactor. Using this approach, the amount of enzyme required by each system can be compared on an equal and fair basis.

## Results and discussion

### Enzyme distribution between solid and liquid phases

**Fig. 2:**
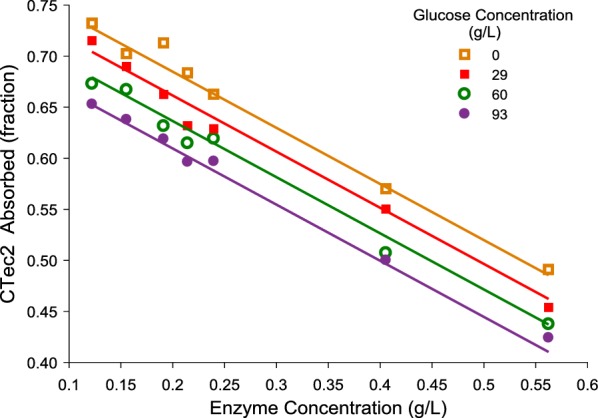
Effects of glucose and enzyme concentrations on the fraction of enzyme absorbed and fitted with Eq. 11. Experimental data are presented by the markers and the optimal fit by the solid lines

Figure 2 shows the effects of glucose and enzyme concentrations on the fraction of enzyme absorbed. As shown in this figure, additional glucose negatively affects enzyme absorption, which is consistent with the literature [24]. As glucose concentration increases, more enzymes combine with glucose in the liquid, thus reducing the enzyme absorbed onto the solid phase. Also, as expected, higher enzyme loadings favor lower fractions of absorption because high dosage might saturate the adsorption of enzymes on the cellulose surface.

To describe the experimental data, a linear equation (Eq. 11) was proposed and resulted in a high coefficient of determination (*R*^2^ = 0.99). This equation was incorporated into the simulation of countercurrent saccharification to quantitatively determine the amount of enzymes in liquid and solid phases, thereby acquiring the transfer amounts and directions of enzymes.

11 $$y = d_{1} \left[ E \right] + d_{2} \left[ {G_{1} } \right] + d_{3}$$

where *y* is the fraction of CTec2 absorbed; *d*_1_, *d*_2_, and *d*_3_ are the parameters (*d*_1_= − 0.550 L/g; *d*_2_ = − 8.04 × 10^−4^ L/g; *d*_3_= 0.795).

### Verification of the CPDM model

**Fig. 3:**
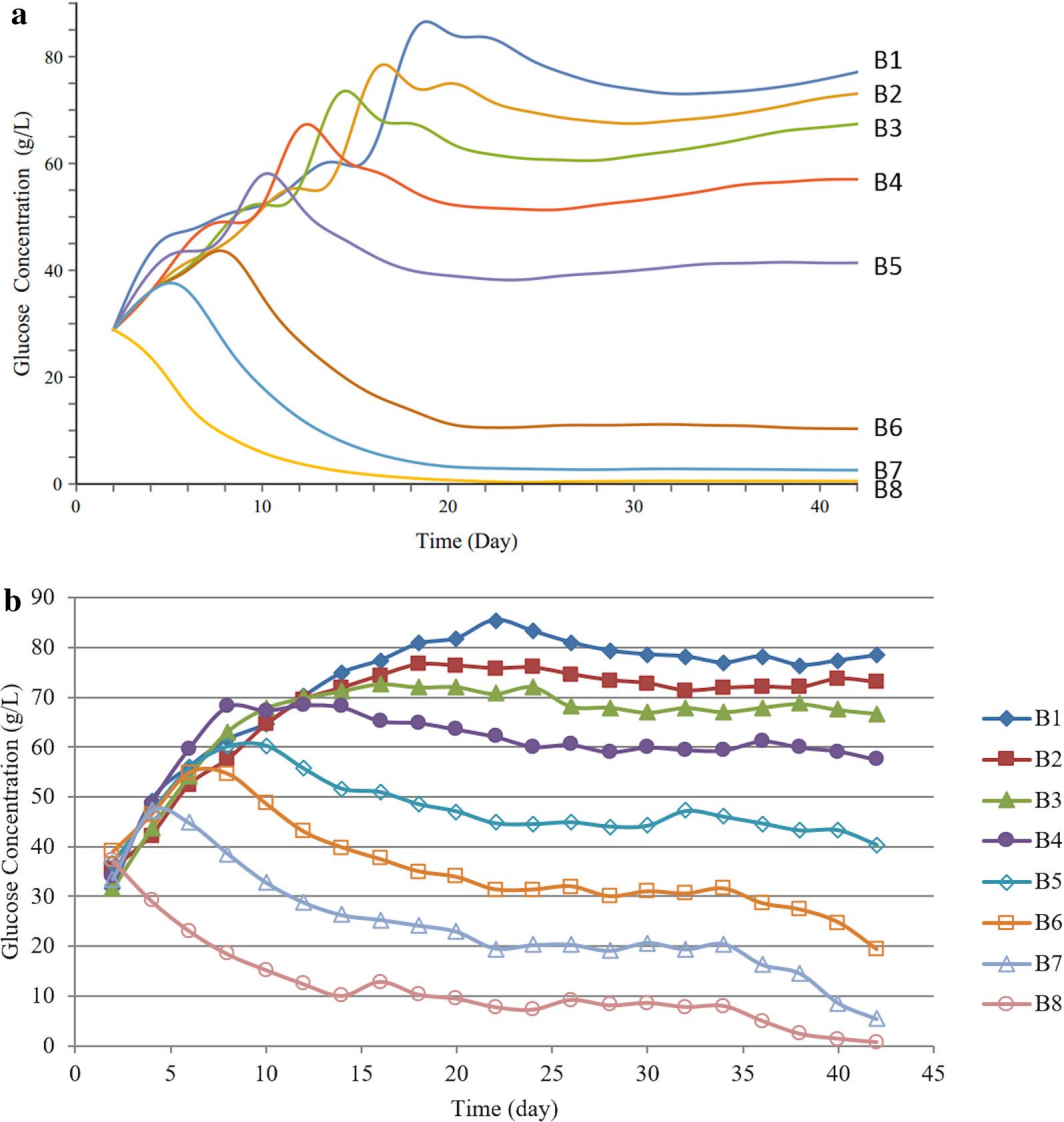
Glucose concentration as a function of time and stage (bottle) number **a** simulation with CPDM **b** experimental data from Zentay et al. [8]. Operation conditions in the simulation are set to be the same as the experimental conditions (listed in Table 1, Train 2)

Figure 3a, b shows the predicted and experimental glucose concentrations as a function of time and stage number at enzyme loading of 5 mg/g. The predicted and experimental results show similar trends. At the beginning, the glucose concentration significantly changes until it eventually stabilizes when the system reaches steady state. At steady state, the glucose concentration increases gradually from Stage 8 to Stage 1. Table 2 compares the experimental glucose concentrations (Stage 1) and conversions to the CPDM predictions at enzyme loadings of 2 and 5 mg/g. To be consistent with experiments, the operation time of the two trains in the simulation was set to be 24 and 42 days, respectively, when the systems have been verified to reach steady state in the experiments. According to Table 2, the CPDM predictions agree well with glucose concentrations from countercurrent experiments with an average error of 3.5%. The average error between experimental and predicted conversions is 4.7%.

**Table 2:** Comparison of experimental and predicted glucose concentrations and conversions for countercurrent saccharification of α-cellulose

Train	1	2	Average
Enzyme loading (mg/g)	2	5	
Glucose concentration (g/L)			
Experimental^a^	54	78	
Predicted from CPDM	51	77	
Error (%)^b^	5.6	1.3	3.5
Conversion (%)			
Experimental^a^	56	88	
Predicted from CPDM	52	86	
Error (%)^b^	7.1	2.3	4.7

^a^Data obtained from Zentay et al. [8]

^b^Error (%) = |Predicted − Experimental|/Experimental × 100%

### Sensitivity analysis

**Fig. 4:**
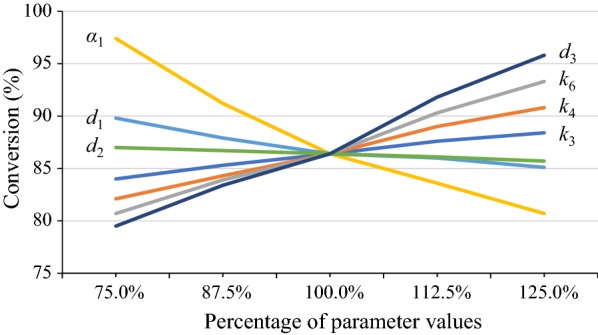
Sensitivity analysis of countercurrent saccharification simulation. Operation conditions in the simulation are set to be the same as the experimental conditions (listed in Table 1, Train 2)

To explore the controlling parameters in the proposed model, sensitivity analyses were performed. Liang et al. [22] showed that *k*_3_, *k*_4_, *k*_6_, and *α*_1_ had the most influence on the modified HCH-1 model results. Therefore, the sensitivities of the four parameters together with the three parameters *d*_1_, *d*_2_, and *d*_3_ in the enzyme distribution equation (Eq. 11) were analyzed in this section (Fig. 4).

As shown in Fig. 4, in the space of 75–125% of parameter values, *α*_1_ and *d*_3_—which are both related to enzyme adsorption—have the most influence on conversion. Among the parameters related to kinetics, *k*_6_—which is considered as the rate constant for recalcitrant cellulose [22]—has the most influence on conversion. The parameter *d*_2_—which weighs the effect of glucose concentration on the fraction of enzyme adsorbed—has the least influence on simulation results. These analyses provide insights to directions that would further optimize the countercurrent system.

### Predictions from the CPDM model

According to the verification results, CPDM is sufficiently accurate to determine the optimal operating conditions in countercurrent saccharification. In this section, CPDM was used to test the effects of enzyme-addition location, total stage number, enzyme loading, LRT, and SLR on conversion and product concentration. To ensure the simulated countercurrent systems reach steady state, the operation time of all simulations was set to 200 days.

#### Effect of enzyme-addition location

**Fig. 5:**
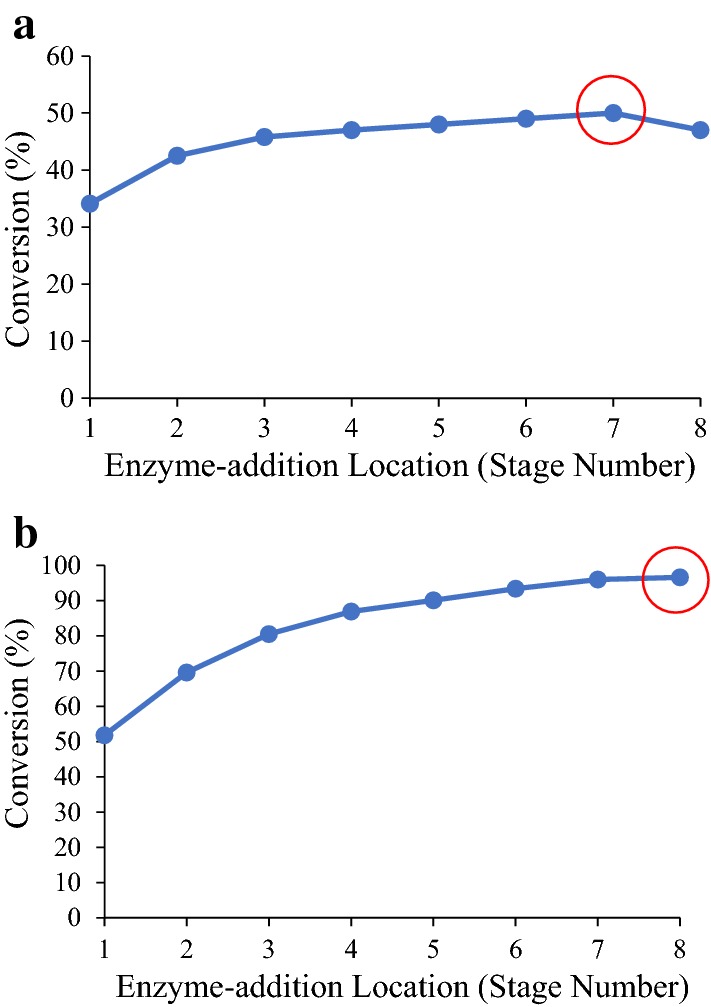
Effect of enzyme-addition location on conversion in the eight-stage countercurrent system at enzyme loadings of **a** 2 mg/g and **b** 5 mg/g using α-cellulose as substrate. Operation time is 200 days. Other conditions in this simulation are set to be the same as the experimental conditions. Simulation data are presented by the markers and the lines are added to show the changing trend

Determining the optimal enzyme-addition location maximizes the retention time of enzymes in the system and therefore fully uses substrate and enzymes. In this simulation study, various enzyme-addition locations were tested in the eight-stage countercurrent system at enzyme loadings of 2 and 5 mg/g (Fig. 5). According to Fig. 5, for both tested enzyme loadings, conversions increase as the enzyme-addition location moves from “front” to “back” of the system. For enzyme loading of 2 mg/g, the highest conversion is obtained when adding enzymes to Stage 7. For enzyme loading of 5 mg/g, the optimal addition location is Stage 8. Compared to low enzyme dosages, at high enzyme dosages, adding enzymes to the downstream brings more benefits. For example, at 2 mg/g, Conversion_Stage 7_ − Conversion_Stage 1_ = 16%. In contrast, at 5 mg/g, Conversion_Stage 8_ − Conversion_Stage 1_ = 45%. At high enzyme dosages, a larger proportion of enzymes are in the liquid phase (“Enzyme distribution between solid and liquid phases” section); therefore, adding enzymes near the “back” of the system increases enzyme retention.

#### Effect of total stage number

**Fig. 6:**
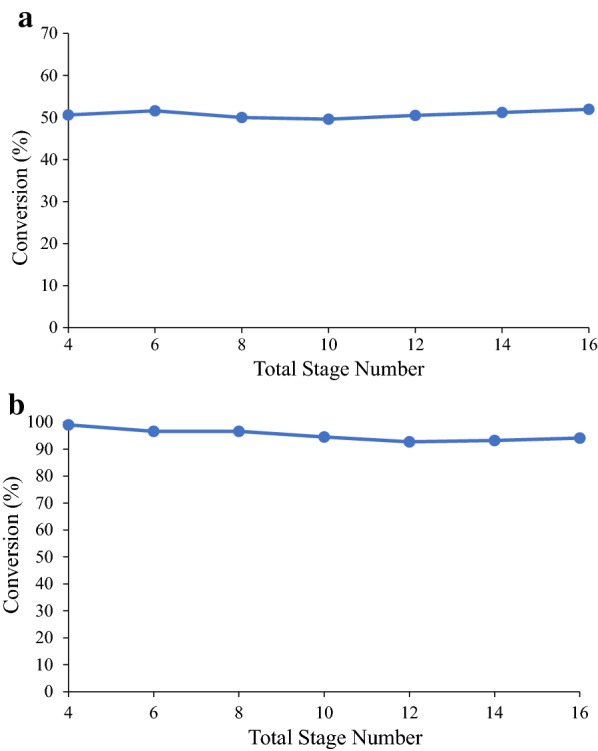
Effect of total stage number on conversion with **a** 2 mg/g and **b** 5 mg/g of CTec2 using α-cellulose as substrate. The enzyme-addition locations are set to be the **a** penultimate stage and **b** last stage. Operation time is 200 days. Other conditions in this simulation are set to be the same as the experimental conditions. Simulation data are presented by the markers and the lines are added to show the changing trend

Figure 6 shows the effect of total stage number on conversion at enzyme loadings of 2 and 5 mg/g. In this simulation study, the transfer frequency was adjusted to ensure the LRTs of all tested conditions were the same (29 days). The enzyme-addition locations were set to the penultimate stage for enzyme loading of 2 mg/g (Fig. 6a) and the last stage for enzyme loading of 5 mg/g (Fig. 6b) (“Effect of enzyme-addition location” section). According to Fig. 6, the optimal total stage number is affected by enzyme loading. For enzyme loading of 2 mg/g, the highest conversion is obtained with a 16-stage system. For enzyme loading of 5 mg/g, the highest conversion is obtained with a four-stage system. This result indicates that for the same LRT, a high-stage system is more beneficial when the enzyme loading is low. In the later simulations, various enzyme loadings were used; therefore, to make a fair comparison, a constant eight-stage system was used.

#### Effect of enzyme loading

**Fig. 7:**
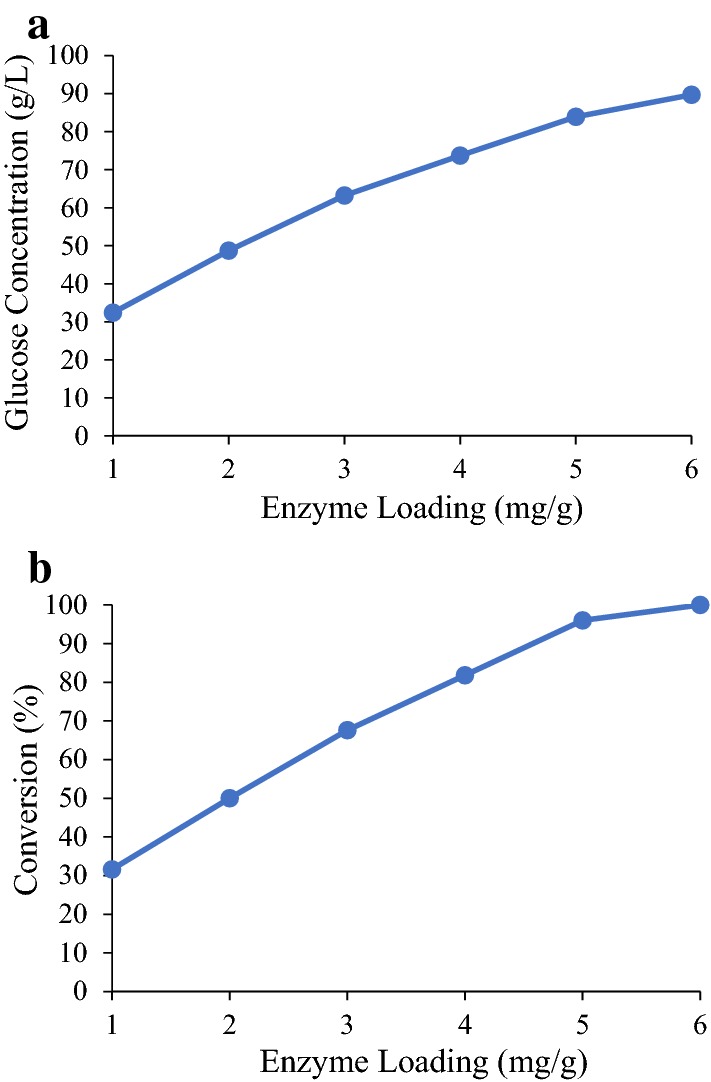
Effect of enzyme loading on **a** glucose concentration and **b** conversion in the eight-stage system using α-cellulose as substrate. Enzyme-addition location is Stage 7. Operation time is 200 days. Other conditions in this simulation are set to be the same as the experimental conditions. Simulation data are presented by the markers and the lines are added to show the changing trend

The effect of enzyme loading on glucose concentration and conversion was simulated in the eight-stage countercurrent system (Fig. 7). To make a fair comparison, the same enzyme-addition location (Stage 7) was used for all test points. According to Fig. 7, as expected, both glucose concentration and conversion increase significantly as the enzyme loading increases from 1 to 6 mg/g. When the enzyme loading is 6 mg/g, the glucose concentration is 90 g/L and conversion is nearly 100%.

#### Effect of liquid residence time and solids loading rate

The LRT (Eq. 12) determines how long the liquid remains in the countercurrent system. A longer LRT allows higher product concentration [26, 27]. To obtain various LRTs, in this study, the transfer frequency was adjusted, which corresponds to *t* in Eq. 4.

12 $${\text{LRT}}\; \left( {\text{day}} \right) = \frac{{{\text{total}}\;{\text{liquid}}\;{\text{volume}}\;{\text{in}}\;{\text{the}}\;{\text{system}} \;\left( {\text{L}} \right)}}{{{\text{flowrate}}\;{\text{of}}\;{\text{liquid}}\;{\text{out}}\;{\text{of}}\;{\text{the}}\;{\text{system}} \,\left( {{\text{L}}/{\text{day}}} \right)}}.$$

The SLR (Eq. 13) represents the rate that biomass is added to the system. A lower SLR allows longer solid residence time, which is a measure of how long the solids remain in the countercurrent system. Longer solid residence times increase digestion, and therefore improve conversion [26, 27]. To obtain various SLRs, in this study, during each transfer, the amount of solid feed added to Stage 1 was adjusted, which corresponds to the continuum particles ($\hat{n}\left( x \right)$) added to conversion = 0 interval in Stage 1.

13 $${\text{SLR}} \left( {{\text{g}}/\left( {{\text{L}}\;{\text{day}}} \right)} \right) = \frac{{{\text{solids}}\;{\text{fed}}\;{\text{per}}\;{\text{day}}\; \left( {{\text{g}}/{\text{day}}} \right)}}{{{\text{total}}\;{\text{liquid}}\;{\text{volume}}\;{\text{in}}\;{\text{the}}\;{\text{system}}\; \left( {\text{L}} \right)}}.$$

**Fig. 8:**
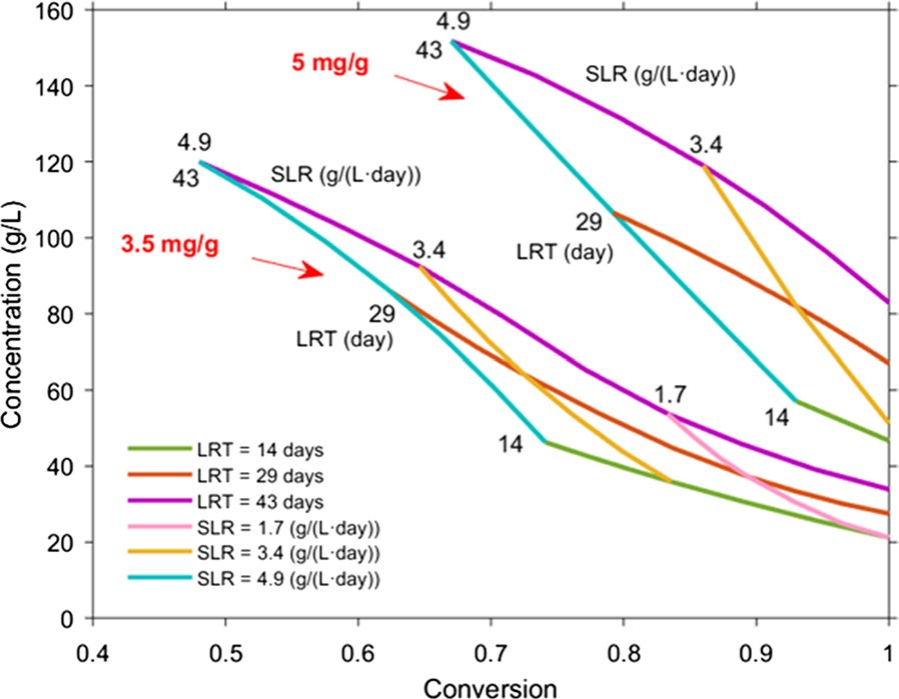
CPDM “map” for countercurrent saccharification of α-cellulose at enzyme loadings of 3.5 and 5 mg/g. Solid concentration in the reactors is 124 g solids/L liquid. Enzyme-addition location is Stage 8. Operation time is 200 days. *LRT* liquid residence time, *SLR* solids loading rate

Figure 8 shows the CPDM “map” for countercurrent saccharification of α-cellulose at enzyme loadings of 3.5 and 5 mg/g with various LRTs and SLRs. The solid concentration in the reactors is 124 g solids/L liquid (0.11 g solids/g (solids + liquid)), the same as the experimental concentration. As shown in Fig. 8, as LRT increases, glucose concentration increases significantly whereas conversion decreases. As SLR decreases, conversion increases significantly whereas glucose concentration decreases. Both observations are consistent with the previous studies using mixed-culture fermentation [26, 27]. Furthermore, as expected, at every LRT and SLR, using enzyme loading of 5 mg/g obtains a higher glucose concentration and conversion compared to 3.5 mg/g. For enzyme loading of 5 mg/g, the “map” predicts a glucose concentration of 152 g/L and a conversion of 67% at LRT of 43 days and SLR of 4.9 g/(L day). A glucose concentration of 83 g/L and conversion of 100% can be obtained at LRT of 43 days and SLR of 2.2 g/(L day). A relatively high glucose concentration (> 100 g/L) and high conversion (> 90%) can be obtained at LRT of 43 days and SLR of 3 g/(L day).

**Fig. 9:**
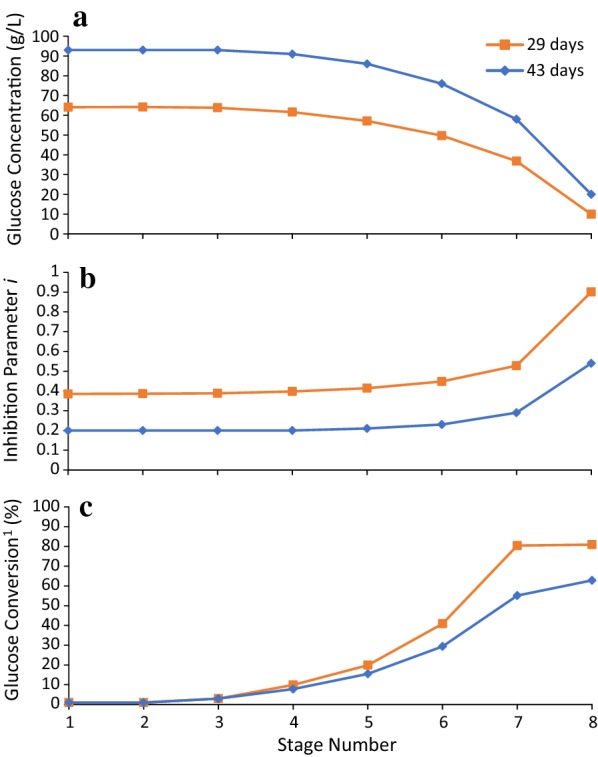
Effect of liquid residence time on **a** glucose concentration, **b** inhibition parameter *i*, and **c** conversion of each stage. Substrate is α-cellulose. Solid concentration in the reactors is 124 g solids/L liquid. Enzyme-addition location is Stage 7. Enzyme loading is 3.5 mg/g. Solids loading rate is 3.4 g/(L day). Operation time is 200 days. $^1 {\text{Conversion}}\;{\text{of}}\;{\text{each}}\;{\text{stage}} \left( \% \right) = \frac{1}{{n_{0} }}\mathop \smallint \nolimits_{0}^{1} x\hat{n}\left( x \right){\text{d}}x \times 100\%$

**Fig. 10:**
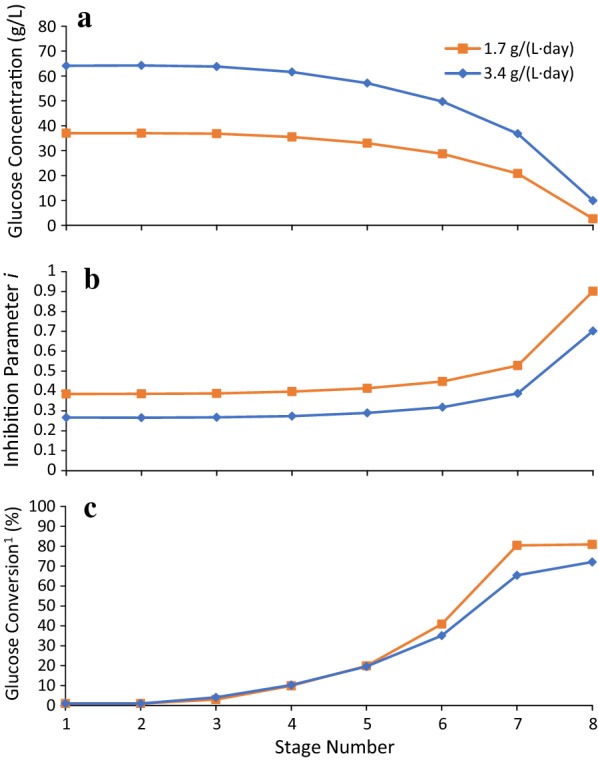
Effect of solids loading rate on **a** glucose concentration, **b** inhibition parameter *i*, and **c** conversion of each stage. Substrate is α-cellulose. Solid concentration in the reactors is 124 g solids/L liquid. Enzyme-addition location is Stage 7. Enzyme loading is 3.5 mg/g. Liquid residence time is 29 days. Operation time is 200 days. $^1{\text{Conversion}}\;{\text{of}}\;{\text{each}}\;{\text{stage}} \,\left( \% \right) = \frac{1}{{n_{0} }}\mathop \smallint \nolimits_{0}^{1} x\hat{n}\left( x \right){\text{d}}x \times 100\%$

Figures 9 and 10 show the effect of SLR and LRT on glucose concentration, inhibition parameter *i*, and conversion of each stage. For 3.5 mg/g added to Stage 7, when using LRT of 43 days, SLR of 3.4 g/(L day), and solid concentration of 124 g solids/L liquid, the obtained glucose concentration of Stage 1 is 93 g/L (Fig. 9a). When decreasing the LRT to 29 days and keeping SLR and solid concentration constant, the obtained glucose concentration of all stages decreases; the glucose concentration of Stage 1 decreases to 64 g/L.

The inhibition parameter *i* relates to glucose concentration only and represents the fraction of total enzyme that is not inhibited. At 93 g/L of glucose concentration (LRT: 43 days, Stage 1), only 20% of enzymes remain active (Fig. 9b). Figure 9b, c shows that as the inhibition parameter *i* increases, the conversion in Stages 5–8 increases significantly. (Note: the conversion of each stage is calculated using particle conversion distribution function: ${\text{Conversion}}\;{\text{of}}\;{\text{each}}\;{\text{stage}} \; \left( \% \right) = \frac{1}{{n_{0} }}\mathop \smallint \nolimits_{0}^{1} x\hat{n}\left( x \right){\text{d}}x \times 100\% ,$ which is different from the previously mentioned conversion for the entire reactor train (Eq. 1)). Similar patterns are shown when increasing SLR from 1.7 to 3.4 g/(L day) and keeping LRT (29 days) and solid concentration (124 g solids/L liquid) constant (Fig. 10).

### Comparison of countercurrent to batch

**Fig. 11:**
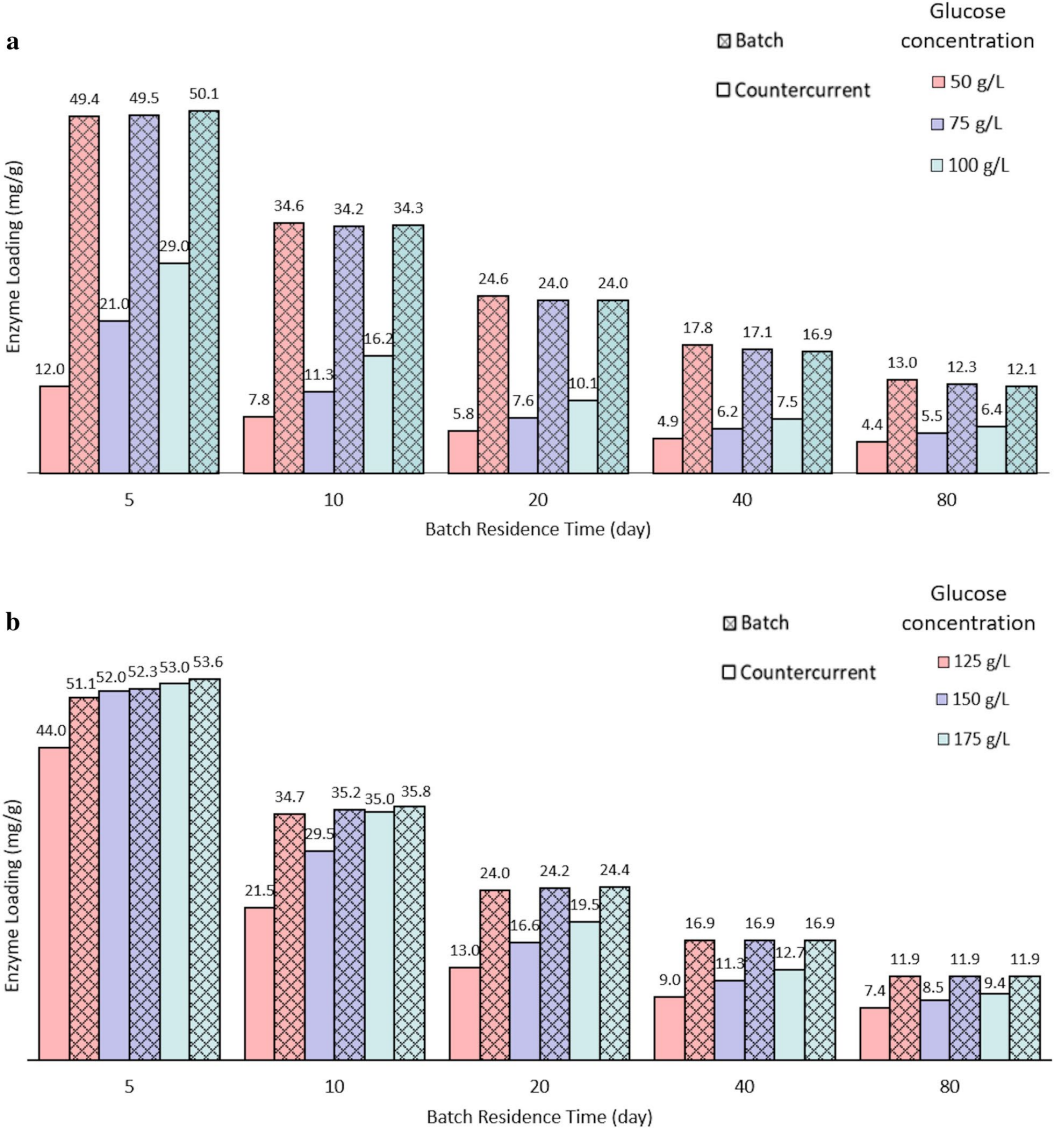
Comparison of enzyme requirements for batch and countercurrent saccharifications at various batch residence time and glucose concentrations, **a** low glucose concentrations and **b** high glucose concentrations. The conversion of all conditions is 100%. Solid concentration in batch (g solids/L liquid) = solid:liquid ratio added to the reactor train (g added solids/L added liquid) in every countercurrent transfer. The liquid residence time in countercurrent saccharification is adjusted to reach the same batch reactor volume using the method in “Reactor volume calculation” section. The solid concentration in every stage in countercurrent saccharification is 250 g solids/L liquid. Enzyme-addition location is Stage 8. Operation time is 200 days. The substrate is α-cellulose. Batch simulations use the modified HCH-1 model. Countercurrent simulations use the CPDM model with the modified HCH-1 equation as the governing equation (Note: in this section, sampling was not included in the simulation of countercurrent saccharification)

To evaluate the efficacy of countercurrent saccharification, an eight-stage countercurrent system is compared with batch saccharification (Fig. 11). Batch simulations use the modified HCH-1 model, and countercurrent simulations use the CPDM model with the modified HCH-1 equation as the governing equation. To compare the enzyme requirement on an equal basis, in this section, batch and countercurrent saccharifications have the same:

1. Conversion: Total conversion (100%) was used.
2. Product concentration: In countercurrent saccharification, the sugar concentration in the product is based on the solid:liquid ratio added to the reactor train; therefore, the batch solid concentration (g solids/L liquid) was set equal to the solid:liquid ratio added to the reactor train (g added solids/L added liquid) in every countercurrent transfer.
3. Reactor volume: This ensures the same capital cost. Using the method in “Reactor volume calculation” section, the LRT in countercurrent saccharification is adjusted to reach the same reactor volume as batch saccharification.

Some industrial reactors, such as percolation reactors, allow for high solid concentrations, which reduces capital costs. In this section, the solid concentration in the reactors in countercurrent saccharification was 250 g solids/L liquid (0.2 g solids/g (solids + liquid)) and the enzyme-addition location was Stage 8.

As shown in Fig. 11, to reach 50 g glucose/L, using 5-day batch residence time, the countercurrent system reduces enzyme loadings by 4.1 times compared to batch; however, the enzyme requirement is still more than 10 mg/g. As batch residence time increases, as expected, the enzyme requirements of all conditions decrease significantly. To reach 50 g glucose/L, at batch residence times of 40 and 80 days, the enzyme loadings of countercurrent saccharification are 4.9 and 4.4 mg/g, respectively, which reduce enzyme loadings by 3.6 and 3 times compared to batch. To reach 100 g glucose/L, at batch residence times of 40 and 80 days, the enzyme loadings of countercurrent saccharification are 7.5 and 6.4 mg/g, respectively, which reduce enzyme loadings by nearly 2 times compared to batch (Fig. 11a). To reach 125 g glucose/L, at batch residence time of 40 days, the enzyme loading of countercurrent saccharification is 9 mg/g, which reduces enzyme loading by 1.9 times compared to batch. To reach 125, 150, and 175 g glucose/L, at batch residence time of 80 days, the enzyme loadings of countercurrent saccharification are all less than 10 mg/g, which reduce enzyme loadings by more than 1.25 times compared to batch (Fig. 11b).

These results indicate that under all conditions, countercurrent saccharification requires less enzymes than batch saccharification; however, it is particularly effective at low product concentrations. In the countercurrent system, when product concentrations are high, the glucose concentrations in the first several stages are all high (such as Fig. 9a). At high product concentrations, enzymes are highly inhibited and the benefits of countercurrent saccharification are less pronounced.

## Conclusions

This study reports kinetic modeling of countercurrent saccharification. The CPDM model was used to simulate multi-stage countercurrent saccharification of α-cellulose with the modified HCH-1 model as the governing equation. This model predicted the experimental glucose concentration and conversion with average errors of 3.5% and 4.7%, respectively, which is sufficiently accurate to determine optimal operating conditions with α-cellulose. CPDM prediction results showed that enzyme-addition location, enzyme loading, LRT, and SLR significantly affected the glucose concentration and conversion. Compared to batch saccharification at the same conversion, product concentration, and reactor volume, countercurrent saccharification is more beneficial when the product concentrations are low.

This study provides a foundation for simulating countercurrent saccharification using the real-world lignocellulose as a substrate. However, because of the complicated composition and structure of lignocellulose, more factors must be considered in future models, such as lignin–enzyme interaction.

## Data Availability

All data generated or analyzed in the present study are included in this article.

## Abbreviations

CPDM: Continuum Particle Distribution Model
CSTR: continuous stirred-tank reactor
PFR: plug-flow reactor
LRT: liquid residence time
SLR: solids loading rate
mg/g: mg protein added/g dry biomass added
DI water: deionized water
